# Supplementary material for: Ixodes ricinus tick bacteriome alterations based on a climatically representative survey in Hungary
Source: Microbiol Spectr. 2023 Nov 15;11(6):e01243-23. doi: 10.1128/spectrum.01243-23 (PMC10715062; doi:10.1128/spectrum.01243-23)
Supplement: Fig. S1 — Bacterial genus abundance correlations. [file spectrum.01243-23-s0001.pdf]

Ixodes ricinus tick bacteriome alterations based on a climatically representative survey in Hungary

Adrienn Gréta Tóth, Róbert Farkas, Marton Papp, Oz Kilim, Haeun Yun, Laszló Makrai, Gergely Maróti, Mónika Gyurkovszky, Eszter Krikó, and Norbert Solymosi

Supplementary information

Species in core bacteriome

Arsenophonus endosymbiont of Aphis craccivora, *A. endosymbiont of Apis mellifera*, *A. nasoniae*.*Bacillus frigoritolerans*, *B. cereus*, *B. mycoides*, *Bacillus* sp. 7D3, *B. thuringiensis*, *B. wiedmannii*.*Candidatus Midichloria mitochondrii*.*Curtobacterium flaccumfaciens*, *Curtobacterium* sp. 24E2, *Curtobacterium* sp. BH-2-1-1, *Curtobacterium* sp. C1, *Curtobacterium* sp. TC1.*Cutibacterium acnes*, *C. avidum*, *C. granulosum*, *C. modestum*.*Mycobacteroides [Mycobacterium] stephanolepidis*, *M. abscessus*, *M. chelonae*, *M. immunogenum*, *M. salmoniphilum*, *M. saopaulense*.*Pseudomonas aeruginosa*, *P. alcaligenes*, *P. antarctica*, *P. azotoformans*, *P. brenneri*, *P. cichorii*, *P. congelans*, *P. eucalypticola*, *P. extremorientalis*, *P. fluorescens*, *P. frederiksbergensis*, *P. graminis*, *P. lurida*, *P. moraviensis*, *P. orientalis*, *P. poae*, *P. prosekii*, *P. putida*, *P. qingdaonensis*, *P. rhizosphaerae*, *P. soli*, *Pseudomonas* sp. 15A4, *Pseudomonas* sp. CIP-10, *Pseudomonas* sp. DG56-2, *Pseudomonas* sp. HN2, *Pseudomonas* sp. HN8-3, *Pseudomonas* sp. LBUM920, *Pseudomonas* sp. NS1(2017), *Pseudomonas* sp. OE 28.3, *Pseudomonas* sp. S49, *P. stutzeri*, *P. synxantha*, *P. syringae*, *P. tensinigenes*, *P. trivialis, P. umsongensis*.*Rhodococcus erythropolis*, *R. fascians*, *R. globerulus*, *R. koreensis*, *R. opacus*, *R. qingshengii*, *Rhodococcus* sp. AQ5-07, *Rhodococcus* sp. B7740, *Rhodococcus* sp. H-CA8f, *Rhodococcus* sp. MTM3W5.2, *Rhodococcus* sp. P1Y, *Rhodococcus* sp. PBTS 1, *Rhodococcus* sp. YL-1. *R. amblyommatis*, *R. asiatica*, *R. bellii*, *R. conorii*, *R. endosymbiont of Ixodes scapularis*, *R. helvetica*, *R. monacensis*, *R. parkeri*, *R. rhipicephali*.*Sphingomonas aliaeris*, *S. alpina*, *S. insulae*, *S. koreensis*, *S. melonis*, *S. paucimobilis*, *S. sanguinis*, *S. sanxanigenens*, *Sphingomonas* sp. AAP5, *Sphingomonas* sp. FARSPH, *Sphingomonas* sp. HMP9, *Sphingomonas* sp. LK11, *Sphingomonas* sp. LM7, *Sphingomonas* sp. NIC1, *Sphingomonas* sp. PAMC26645, *S. taxi*.*Staphylococcus arlettae*, *S. aureus*, *S. auricularis*, *S. capitis*, *S. epidermidis*, *S. haemolyticus*, *S. hominis*, *S. pasteuri*, *S. saccharolyticus*, *S. warneri*.*Stenotrophomonas acidaminiphila*, *S.* indicatrix, *S.* maltophilia, *S.* rhizophila, *Stenotrophomonas* sp. 169, *Stenotrophomonas* sp. DR822, *Stenotrophomonas* sp. LM091, *Stenotrophomonas* sp. NA06056, *Stenotrophomonas* sp. SI-NJAU-1, *Stenotrophomonas* sp. SXG-1.*Wolbachia endosymbiont of Anopheles demeilloni*, *W. endosymbiont of Ceratosolen solmsi*, *W. endosymbiont of Chrysomya megacephala*, *W. endosymbiont of Corcyra cephalonica*, *W. endosymbiont of Delia radicum*, *W. endosymbiont of Drosophila simulans*, *W. endosymbiont of Wiebesia pumilae*, *W. pipientis*.

Pathogens

Taxon classification of short reads with Kraken2 resulted in hits for the following pathogenic bacteria. *Anaplasma phagocytophilum* was found in nymphs from sampling sites 3 and 7. Reads from *Borrelia coriaceae* were identified in females collected from sampling site 1. *B. miyamotoi* was found in females from sampling sites 2 and 16 and in nymphs collected from sampling sites 1 and 10. *Borreliella garinii* was at sampling site 11 in the nymphs. *B. valaisiana* was found in nymphs collected at sampling site 8 and 11. *B. afzelii* was found at sampling site 2 in nymphs. *Ehrlichia muris* related reads were found in female 12 and nymph 14 samples. Reads originating from the genus *Rickettsia* were found in all samples: *R. amblyommatis* in females (sample site: 5) and nymphs (sample site: 6); *R. asiatica* in females (1, 7, 12) and nymphs (1, 2, 10, 15); *R. bellii* in females (5) and nymphs (6, 10); *R. conorii* in females (8); *R. helvetica* in females (1, 3-5, 7-10, 13-17) and, nymphs (1-6, 8, 10, 11, 13-17); *R. monacensis* in females (3, 5, 8) and nymphs (1, 6, 10, 12); *R. parkeri* in nymphs (10); *R. rhipicephali* in females (5, 8) and nymphs (1, 6, 10). No species from the genera *Bartonella*, *Coxiella* and *Francisella* was found.

The result of the BLAST based taxon classification of the assembly-generated contigs is as follows: *Candidatus Odyssella thessalonicensis* L13 was found in females (sample site: 5); *Candidatus Rickettsia colombianensi* in females (5, 8, 15) and nymphs (4, 6, 10, 13); *Orientia tsutsugamushi* in females (5) and nymphs (10); *Rickettsia akari* in nymphs (6, 10); *R. asembonensis* in females (5, 8, 17) and nymphs (6, 8, 10, 13); *R. asiatica* in females (3, 4, 5, 7, 8, 14, 15, 16, 17) and nymphs (1, 2, 3, 4, 6, 8, 10, 11, 13, 14, 15, 16, 17); *R. australis* in females (5, 8, 15) and nymphs (3, 4, 6, 10, 11, 14); *R. bellii* in females (8) and nymphs (1, 6, 10); *R. canadensis* in females (3, 5, 7, 8, 14, 17) and nymphs (1, 3, 6, 10, 11, 15); *R. conorii* in females (5) and nymphs (6, 10, 15); *R. felis* in females (1, 5, 7, 8, 13, 15, 17) and nymphs (1, 3, 4, 6, 10, 11, 14, 15); *R. fournieri* in females (4, 5, 8, 15) and nymphs (1, 4, 5, 6, 10, 11, 15); *R. gravesii* in females (5, 8) and nymphs (3, 6, 10); *R. helvetica* in females (1, 3, 4, 5, 7, 8, 13, 14, 15, 17) and nymphs (1, 2, 3, 6, 8, 10, 11, 13, 14, 15, 16); *R. honei* in females (5, 7, 8, 14, 17) and nymphs (3, 6, 10); *R. hoogstraalii* in females (4, 5, 8, 15, 17) and nymphs (1, 3, 6, 10, 15); *R. japonica* in females (5) and nymphs (6, 10); *R. monacensis* in females (5, 8, 15) and nymphs (1, 4, 6, 10); *R. prowazekii* in females (5, 8, 14, 15) and nymphs (1, 6, 10, 11); *R. rhipicephali* in females (1, 3, 5, 8) and nymphs (1, 3, 6, 10, 11); *R. rickettsii* in females (5) and nymphs (6, 10); *R. sibirica* in females (5) and nymphs (6); *R. slovaca* in females (1) and nymphs (6, 10, 15); *Rickettsia* sp. MEAM1 in females (5, 7, 8, 15) and nymphs (6, 10, 11); *R. tamurae* in females (4, 5, 7, 8, 15, 17) and nymphs (1, 4, 6, 10, 13, 14, 15); *R. tillamookensis* in females (4, 5, 17) and nymphs (1, 2, 3, 6, 10, 11, 15); *R. typhi* in females (8) and nymphs (4, 6, 10); *Spiroplasma endosymbiont of Danaus chrysippus* in females (13).

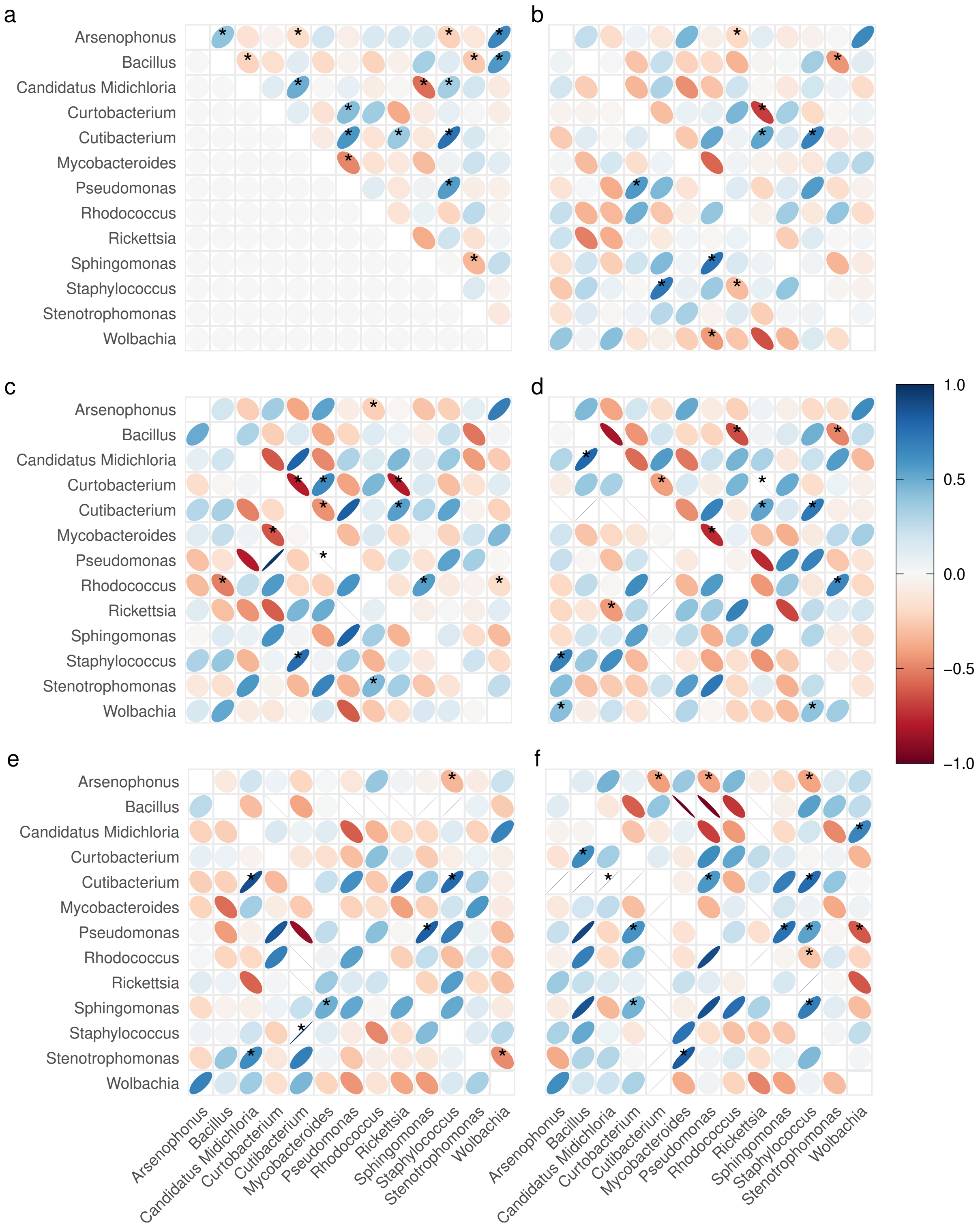

Fig S1. Bacteria genera abundance correlations. The correlation in all ticks is shown in Figure a. The lower half of f gure b is obtained in nymphs and the upper half in females. In f gure c, the correlations in females from cooler environments are in the lower, and those from warmer environments are in the upper triangle. In f gure d, the correlations for females from drier environments are in the lower triangle and those from the wetter environment in the upper one. In f gure e, the correlations of nymphs from cooler environments are in the lower triangle, and those from warmer environments are in the upper triangle. In f gure f, the correlations of the nymphs from drier environments are in the lower triangle, and those from a wetter environment are in the upper triangle. Signif cant ($p<0.05$) relationships are marked by *.
